# Are Age and Trauma Mechanism Associated with Volume Change in the Fractures of the Bony Orbit?

**DOI:** 10.3390/jcm13123618

**Published:** 2024-06-20

**Authors:** Ella Starck, Niilo Lusila, Juho Suojanen, Eeva Kormi

**Affiliations:** 1 Clinicum, Faculty of Medicine, University of Helsinki, 00014 Helsinki, Finland; 2 Päijät-Häme Joint Authority for Health and Wellbeing, Department of Radiology, Päijät-Häme Central Hospital, 15850 Lahti, Finland; 3 Päijät-Häme Joint Authority for Health and Wellbeing, Department of Oral and Maxillofacial Surgery, Päijät-Häme Central Hospital, 15850 Lahti, Finland; 4 Cleft Palate and Craniofacial Centre, Department of Plastic Surgery, Helsinki University Hospital, 00029 Helsinki, Finland

**Keywords:** orbit, blowout fracture, blow-out fracture, orbital volume, facial trauma, automated segmentation

## Abstract

Blowout fractures are common midfacial fractures in which one or several of the bones of orbital vault break. This is usually caused by a direct trauma to the eye with a blunt object such as a fist. Fracturing of the fragile orbital bones can lead to changes in the orbital volume, which may cause enophthalmos, diplopia, and impaired facial aesthetics. **Objectives**: The aim of this study is to investigate whether there is an association between volume change of the bony orbit and age, gender, or trauma mechanism. **Methods**: A retrospective study of patients with unilateral blowout or blow-in fractures treated and examined in Päijät-Häme Central Hospital, Lahti, Finland was conducted. Altogether, 127 patients met the inclusion criteria. Their computed tomographs (CT) were measured with an orbit-specific automated segmentation-based volume measurement tool, and the relative orbital volume change between fractured and intact orbital vault was calculated. Thereafter, a statistical analysis was performed. A *p*-value less than 0.05 was considered significant. **Results**: We found that relative increase in orbital volume and age have a statistically significant association (*p* = 0.022). Trauma mechanism and gender showed no significant role. **Conclusions**: Patient’s age is associated with increased volume change in fractures of the bony orbit.

## 1. Introduction

Midfacial fractures include fractures in the bony orbit, maxilla, zygoma, and naso-orbitoethmoid (NOE) complex [1]. They can be classified as Le Fort I–III, zygomatic arch fractures, zygomaticomaxillary complex fractures, NOE fractures, and orbital bone fractures occurring either individually or combined [2]. Midfacial trauma is often associated with ocular injuries of varying severity [3]. The AOCMF Classification Group has divided orbital fractures into four groups, as follows: orbitozygomatic fractures, naso-orbitoethmoidal fractures, internal orbital fractures, and combined orbital fractures [4]. Internal orbital fractures, such as blowout and blow-in fractures, include fractures in the floor, the medial and lateral walls, and the roof [5].

The bony part of the orbit consists of seven parts: frontal bone, zygomatic bone, maxilla, lacrimal bone, ethmoid bone, sphenoid bone, and palatine bone, and together they form the four walls of the pyramid-like orbit. The orbital floor is one of the thinnest parts, anatomically weakened by the infraorbital nerve, and therefore, one of the most common parts of the orbit to fracture [6]. Another very thin orbital structure that often gets damaged is the medial wall. According to Hammer, the posterior part of the medial wall is a “key area”, because it, along with the lateral wall, holds the globe in its right position, and it is clinically shown that repairing an orbital fracture with an intact “key area” is technically a lot easier than when this section is also fractured [7].

A blowout fracture is usually caused by a blunt-force trauma, such as a fist, hitting the eye area and causing the surrounding bone structures to fracture. In particular, a fracture of the weak, paper-thin orbital floor often leads to bone fragments and orbital contents sagging into the inferior maxillary sinus [2]. The fractured edges may also cause entrapment of orbital contents [8]. In blow-in fractures, the orbital floor breaks inwards towards the orbit, and it is thus considered a reversed blowout fracture However, blow-in fractures are distinctly more uncommon than blowout fractures [9]. Orbital fractures are classified as pure or impure, depending on whether the orbital rim has remained intact or not, respectively [3]. In blowout and blow-in fractures, the orbital rim remains intact but the fragile inferior section breaks, resulting in a volume change in the bony orbit [8].

There are two main mechanisms for fractures in the bony orbit. According to the hydraulic theory, the retropulsion of the globe increases the infraorbital pressure, which transmits to the orbital walls, causing a fracture. The buckling theory maintains that direct trauma to the rigid orbital rim shifts the force posteriorly and creates a compression fracture of the orbital floor [10]. These two mechanisms, when combined, cause fractures that occupy wider areas of the orbital walls than when occurring separately [11].

Among geriatric patients, orbital fractures are more common than in younger controls and most frequently caused by falls on the ground [12]. Moreover, orbital fractures are, remarkably, often more extensive than in younger patients and most commonly affect the middle and posterior parts of the orbital floor [13]. A study by Toivari et al. shows that when the orbit is divided into three parts (the anterior, middle, and posterior thirds), the most common areas to fracture in the elderly are all three thirds, the middle and posterior thirds, and the posterior third, and the difference to younger controls is significant [13].

When treating younger patients, the maturity of the orbit must be considered. In children and teenagers, the orbital roof is the most common frontobasal structure to break and the main actor for this is a high-velocity trauma [14]. An immature facial bone resists greater forces since the growing sutures are not yet ossified and the bones can even deform momentarily because of their more elastic structure [15]. Therefore, in younger patients a more common fracture is the trap door fracture, signifying a linear, slightly dislocated fracture that entraps, typically, the inferior rectus muscle [8].

Fractures in the bony orbit and, hence, orbital volume changes can cause globe malposition, such as enophthalmos or hypo-ophthalmos, and, consequently, diplopia. Entrapment of muscle in the fracture territory may lead to restriction of eye movement, usually in the up and down gaze [3]. The infraorbital nerve passes through the orbital floor and can therefore be injured in orbital fractures. Fracture-related oedema, compression, ischemia, or nerve laceration can also cause nerve damage [16]. This can appear as numbness, paraesthesia, dysesthesia, or anaesthesia in the surrounding area.

The aims of treatment concerning orbital fractures are to restore normal ocular motility to provide binocular vision and to prevent enophthalmos for cosmetic reasons [3]. Surgery is also used to preserve the orbital volume. Due to a lack of evidence, there is still controversy regarding the criteria for surgery. It depends on an overall multidisciplinary clinical examination and an estimation of the severity of the orbital damage, often specified using computed tomography [17]. Most fractures do not need treatment [18]. However, if the decision is to treat the fracture surgically, it should happen within two weeks, though it can be carried out up to four weeks after the trauma with satisfactory results [3]. In many cases, the patient has other more severe injuries that demand immediate treatment, and the tissue oedema caused by the fracture usually worsens within a few days from the injury, making the treatment more difficult and therefore, delaying the fracture repair even more [2].

The most commonly used surgical methods for orbital fractures are transconjunctival, subtarsal, and subciliary incisions, with modifications depending on the location and appearance of concomitant injuries [3]. However, the transconjunctival method is becoming more common because it causes fewer complications than the others and has the most favourable results aesthetically [19]. Regardless of the method used, it is recommended to overcorrect the defect [3]. Complex orbital fracture repair should happen in two phases, starting with the reconstruction of the orbital rim and thereafter, correction of the internal orbit, since the orbital frame guides the correct structure of the internal part [5]. Materials used for orbital floor reconstruction are autogenous grafts such as the iliac crest or calvaria; alloplastic materials, including titanium mesh, polyethylene, polytetrafluoroethylene, and polymeric silicone; or allogenic materials, like lyophilised dura, allogenic bone, and cartilage [3].

Nonoperative treatment of fractures in the bony orbit includes sinus precautions, which include avoiding nose blowing, smoking, and drinking through a straw [20]. Posttraumatic ophthalmic oedema can be treated medically with either topical or systemic corticosteroids [21]. Elevated infraorbital pressure can produce traumatic glaucoma, and should, therefore, be treated. Usually, topical beta-blockers or prostaglandin analogues are used, though the latter have a proinflammatory effect [22,23]. Since infections after an orbital trauma are rare regardless of the use of prophylactic antibiotics, they should not be prescribed [24]. However, if antibiotics are needed to treat ophthalmic infections, topical chloramphenicol is widely used [25]. Additionally, when treating orbital fractures nonoperatively, a specialist should keep track of the situation for a short period of time [26].

Orbital volume can be measured using either automated segmentation tools or by manually tracing and marking the orbital walls in each computed tomography (CT) slice. The marking of walls slice by slice has been a gold standard, but it is relatively time-consuming, and it is also possible that inter- and intra-observatory errors affect the reliability. In this study, orbital volume was measured through automated segmentation with the Bonelogic CMF Orbital programme (Disior, Ltd., Helsinki, Finland). This technique gauges orbital volume by converting the radiographic image to voxels, and thereafter, creates a three-dimensional interpretation of the bone structure with a method based on thresholds. Ultimately, the evaluator indicates the programme about the fractured orbit. The HU range for soft tissues is needed and can be manually adjusted if necessary. Otherwise, the segmentation happens without user interaction [27].

Facial trauma profiles of different age groups have been thoroughly reported, as mentioned above. However, the volumetric changes, especially in non-operated patients, have not been reported substantially, probably due to the amount of manual labour required for segmenting the orbits slice-by-slice from the CT. The aim of this study is to investigate the impact of a patient’s age and trauma mechanism on the relative volume change in fractures of the bony orbit. The hypothesis is that older patients have larger changes in relative orbital volume than younger patients because the bone structure gets more fragile and less elastic with ageing, and younger patients’ bones can withstand higher forces due to the still-maturing bone structure [15,28]. The hypothesis is that certain trauma mechanisms cause greater relative volume changes than others.

## 2. Materials and Methods

A retrospective study of patients with fractures of the bony orbit at Päijät-Häme Central Hospital (PHCH), Lahti, Finland between years 2008 and 2020 was conducted. The study was approved by the institutional review board of PHCH (D/18/07.01.04.05/2018). The participants were searched from PHCH’s patient register using International Classification of Diseases (ICD) codes S02.00, S02.30, S02.40, S02.64, S02.70, S02.8, S05.x, and S06.x, which coincide with fractures and trauma of the skull and facial bones. Inclusion criteria were one-sided fracture of the bony orbit, examined or treated in PHCH, with computed tomography (CT) taken. Only CTs with a slice thickness of 2 mm or less were included in the study. Orbital floor fractures with combined zygomatic complex fractures were excluded. The variables in the study were relative orbital volume change, age, gender, and trauma mechanism. Relative orbital volume change was the outcome of the study; patient’s age was the predictor; and the patient’s gender and trauma mechanism were the confounders. Trauma mechanisms were classified as fall, assault, sports-related accident, traffic-related accident, and other.

**Figure 1:**
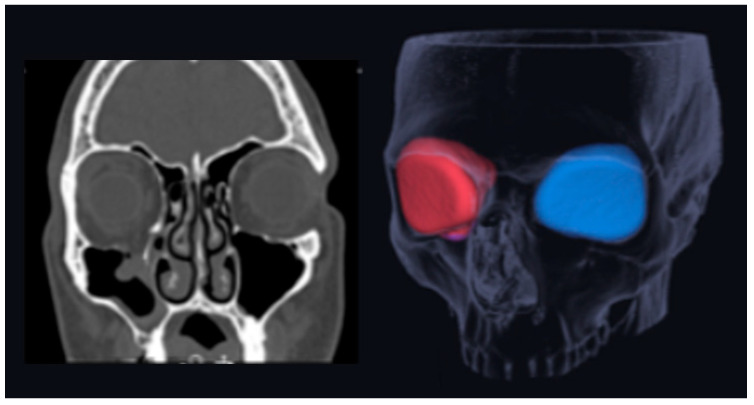
Orbital volume is measured from CT scans with an automated segmentation tool in which a virtual triangle mesh expands from a starting point in the bony orbit to another on the other side. The volume of the fractured orbit (red) is compared to the intact (blue) one to obtain the relative orbital volume change.

After sorting out patients with the ICD diagnosis codes mentioned above, their radiographs were examined to identify patients with blowout or blow-in fractures. The volumes of the intact and the fractured bony orbit were measured with the Bonelogic CMF Orbital program (Disior Ltd., Helsinki, Finland). This automated segmentation tool measures the orbital volume automatically after the radiologist has selected a seed-point inside the orbital vault. In the cases of this study, the apex of the orbit at the conjunction of the optic nerve and bulbus was used. After confirming the side, the virtual triangle mesh starts expanding until it meets the bony walls and the anterior rim of the orbit. This procedure is pointed out in Figure 1. Of the fracture, the shortest distance between the rims of the fragment and floor rims limits the expanding mesh. Thus, in blow-in fractures the area under the fragment is not included in the volume. The reliability of the software measurements is proven to be high (0.992 (95% CI 0.987–0.997 intra-observer ICC, and 0.989 (95% CI 0.983–0.993)) interobserver ICC in the intact orbit) [27]. The relative volume change was calculated by comparing the orbital volume of the injured side to that of the intact contralateral side. The rest of the variables were found and collected from the patient register.

All measurements were made, and the results were controlled by the same radiologist (N.L.) to prevent research bias. In addition, the participants found in the patient register according to certain ICD codes were, thereafter, manually verified. The study size was achieved through a data collection of fractures of the bony orbit over a period of 12 years.

The data were collected using Microsoft Excel software (MS Office 365, year2021 version). For a statistical analysis, the data were transferred to the statistics software SPSS (IBM^®^, version 29). The significance was set to 0.05. We tested the distribution of data with a Shapiro–Wilks test. If the test showed evidence of non-normality, we used a non-parametric test. We compared the relative orbital volume change with sex and trauma mechanism with Mann–Whitney U and Kruskal–Wallis two-sided tests, respectively. For the association of relative orbital volume change with age, Spearman’s rho was utilied. For further analysis, the association of age with trauma mechanism and sex was calculated.

## 3. Results

Overall, 138 patients with blowout or blow-in fractures were obtained from the PCHC patient register, with radiographs meeting inclusion criteria. Of them, 11 patients’ orbital volume could not be measured properly due to inadequate CT data. In consequence, 127 patients were left for final analysis.

Demographic data is shown in Table 1. The continuous variables did not follow a normal distribution. The age ranged between 9.9 and 91.4 years, with a mean of 48.2 years. The average relative volume change in percentage compared to the contralateral side was 4.4%. In some patients, the relative orbital volume had collapsed, and in others, it increased. The change varied between -16.0% and 65.3%, with a mean of 4.39%. Of the 127 patients, 51 were female (40.2%) and 76 were male (59.8%). Trauma mechanisms were classified as assault, fall, sports-related accident, traffic-related accident, and other. Assaults were the cause of fractures in 56 patients (44.1%), falls caused 44 fractures (34.6%), sports accidents 12 (9.4%), traffic accidents 11 (8.7%), and others 4 (3.1%).

**Figure 2:**
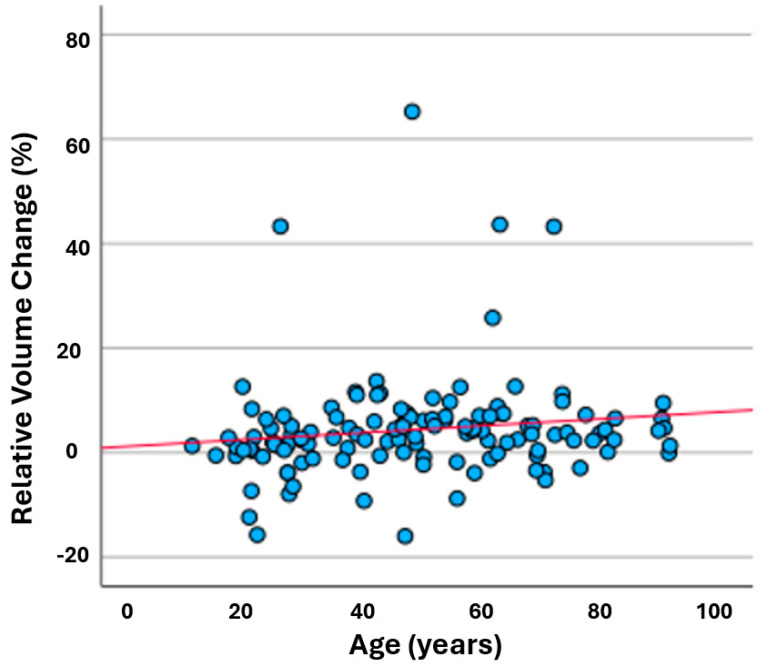
Relative volume change increased along with patient’s age. Each blue dot represents individual patient, and red line shows average relative (%) volume change compared to age.

**Figure 3:**
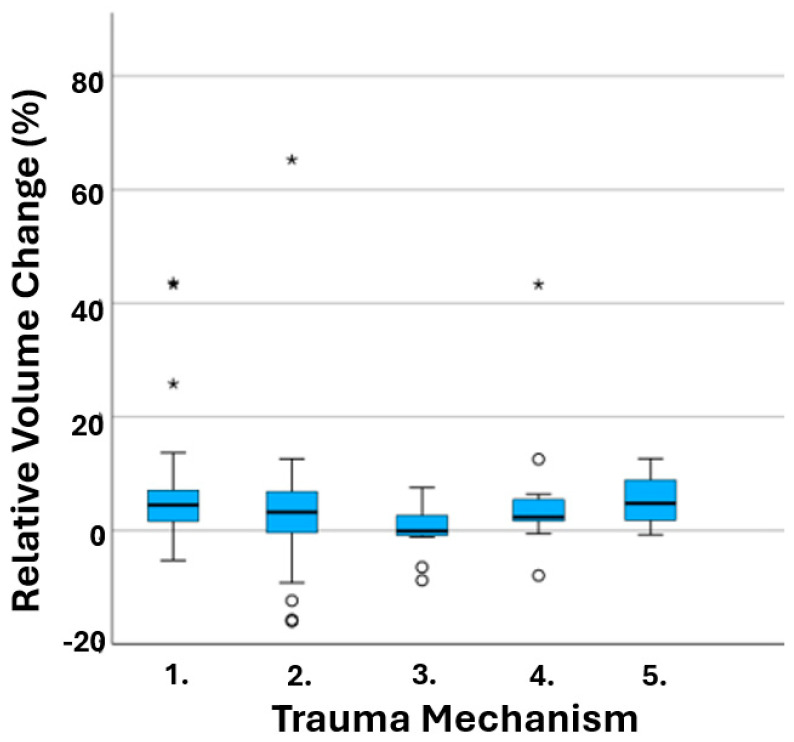
Relative volume change and trauma mechanism have no correlation. Blue box represents 50% of the patients and median; whiskers represent highest and lowest 25 percentages; stars and dots demonstrate the measurement outliers.

The only statistically significant association with relative orbital volume change was the age of the patient (*p* = 0.022), as the relative volume change increased along with an increase in the patient’s age (Figure 2). Trauma mechanism (Figure 3) and sex showed no statistically significant role in relative volume change (*p* = 0.535 and *p* = 0.101, respectively). These can be seen in Table 2.

**Figure 4:**
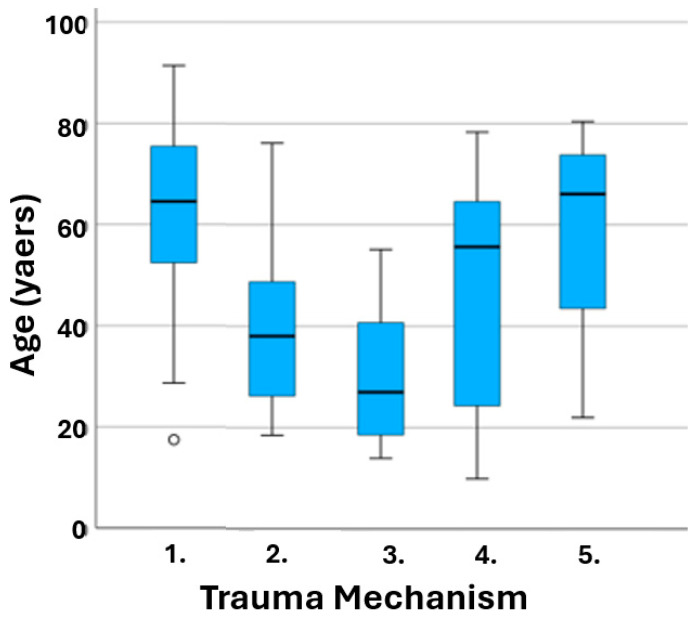
Trauma mechanisms’ association with age: 1. fall, 2. assault, 3. sports, 4. traffic, 5. others. Blue box represents 50% of the patients and median; whiskers represent highest and lowest 25 percentages.

**Figure 5:**
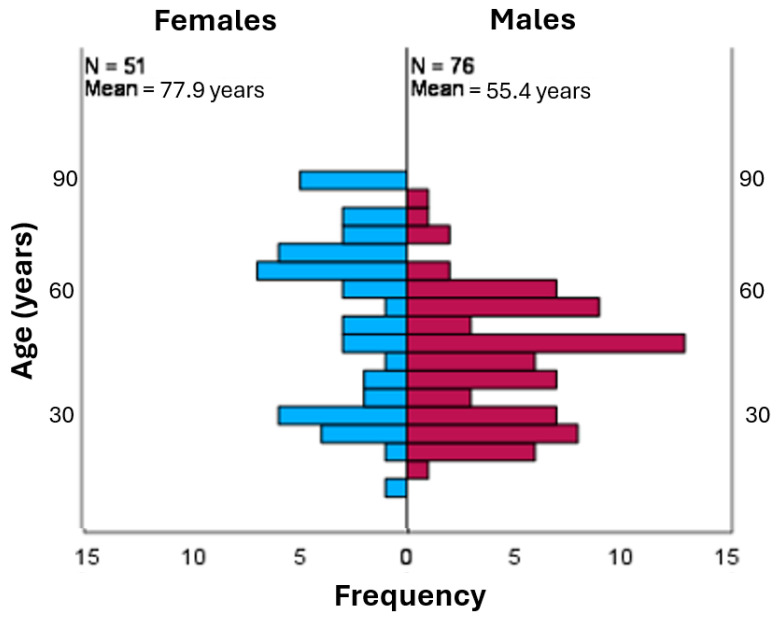
Females and males have distinct age profiles. Females more frequently suffer orbital traumas as young adults and in older age, whereas male are more susceptible to trauma in middle age.

However, gender and trauma mechanism had a significant association with age, as seen in Table 3 and Figure 4. In further analysis of the trauma mechanism, pairwise comparison showed significant difference in sports and fall (*p* = 0.000) and assault and fall (*p* = 0.000). In addition, a significant difference between the patients’ age and gender can be seen in Figure 5.

## 4. Discussion

The aim of this study was to investigate the impact of a patient’s age and trauma mechanism on the relative volume change in fractures of the bony orbit. The hypothesis was that older patients have bigger changes in relative orbital volume than younger patients because the bone structure becomes more fragile and less elastic with ageing, and younger patients’ bones can withstand higher forces due to the still-maturing bone structure [15,28].

The average global orbital volume is 24.5 mL, with a range of 16.9–35.0 mL. Males tend to have a larger volume than females, but a slow growth in volume beyond 30 years is also pointed out [29]. Orbital blowout or blow-in fractures often lead to changes in orbital volume. These changes can be measured with three-dimensional computed tomography scans to facilitate treatment planning and damage evaluation. Several methods and software programmes have been developed to measure the orbital volume manually, semiautomatically, or automatically, though manual planimetry is still the most widely used. However, it is important to keep in mind that the orbital volume is strongly dependent on the measurement method used since the orbital borders are not unequivocal [30]. Generally, the unaffected orbit can be utilised to mirror and restore the fractured orbit since the possible asymmetries are statistically minor [31]. However, patients with unilateral cleft lip and palate tend to have asymmetries in the bony orbit, which needs to be recognised in the treatment planning of a fractured orbit [32].

An increase in orbital volume can cause enophthalmos, which is why posttraumatic volume and its correlation with enophthalmos have been examined in various studies. Most of them agree that increased orbital volume correlates highly with the degree of enophthalmos [33,34,35,36]. Controversially, some studies regard enophthalmos as being caused by herniation of orbital contents and soft tissue displacement [37,38]. Some studies have even found that there is correlation between enophthalmos and both herniated tissue and orbital volume change [39]. There are studies about orbital volume changes after fractures in the bony orbit, but these treat, for instance, orbitozygomatic complex fractures [40] and zygomatic fractures [41] and can, therefore, not be compared to our study, in which patients were chosen for only having individual blowout or blow-in fractures.

Oh et al. [36] observed the relative orbital volume change in blowout fractures depending on the fracture site. They found that fractures in the inferior and medial walls caused greater relative volume changes (mean 121.5%) than inferior wall fractures (mean 113.5%), and that fractures in the medial wall had the smallest relative volume change (mean 106.9%) among these three groups. However, the relative volume changes cannot be directly compared with ours since the radiologic volumetric measurement tools that were used are not comparable to those of our present study. It has also been found that larger fractures of the bony orbit, which lead to greater volume changes, are more likely to cause globe malposition [42]. In our study, the relative volume change was more often increasing than decreasing (mean 4.4%). This is supported by earlier studies, which agree that isolated orbital fractures tend to lead to a volume increase [43,44]. Decreasing volume results from the orbit’s bony structures collapsing inwards towards the orbit and is more uncommon since orbital blow-in fractures are remarkably rarer than blowout fractures. Decreased volume changes lower the mean value and, therefore, seem to result in smaller average damage. This can be observed by comparing our mean relative volume change to that of a study by Pancell et al. [44] that examined exclusively nonoperatively treated blowout fractures but still had a higher mean (8.6%), even if nonoperatively treated fractures often tend to have smaller volume changes than the average of all patients with blowout fractures. Operative treatment is not straightforwardly connected to the volume change [45], but is correlated with enophthalmos, which is one of the indicators for surgical treatment [46,47].

As expected, we found that there is a statistically significant correlation between relative orbital volume change and a patient’s age. The older the patient, the greater the relative volume change. There are no earlier studies about this, but Toivari et al. [13] found that geriatric patients (65 years of age or older) are more likely to sustain more extensive fractures than younger ones, which may also correlate with greater relative volume changes. This could be explained by the fact that bone composition varies with age, and cortical bone becomes thinner with age, making it more fragile [28,48]. In addition, Lee et al. [49] concluded that osteoporosis, which is more common among older adults and especially postmenopausal women, makes a remarkable difference in midfacial bone density compared to patients without osteoporosis. Midfacial bones involve the orbital floor, which is, hence, more fragile among patients with osteoporosis.

Our patient material contained only four patients under the age of 18, and therefore, cannot be used for generalised knowledge concerning paediatric treatment. Altogether, midfacial fractures are unusual in young patients, and there is not much information about them [50]. Su et al. [47] examined orbital blowout fractures in underaged patients and found that patients aged between 13 and 18 years had the highest incidence compared to the age groups 0–6 years and 7–12 years. According to them, this is because teenagers become more independent and encounter more conflicts and activities with higher risks for injuries. It has also been stated that orbital fractures become more common when children age because the midfacial part grows in proportion to the rest of the face and is, therefore, especially the orbital floor, more vulnerable to fracture than in younger children [51].

Trapdoor fractures, in which the fractured bone returns to its original place, clasping the orbital content with it, are more common in children and adolescents than in older patients [8,52]. This is explained by the more elastic consistency in younger bones due to the bone structure having more osteocytes than osteoblasts compared to older bone structure [52]. Our patient material included only a few children and adolescents; hence, this cannot be universalised. Nagasao et al. [53] examined the morphology of the orbital floor and its differences with respect to age and gender. They found that the orbital floor’s lowest point proceeds to lower and moves towards the back with age. Also, the floor inclination is steeper in children and males. All in all, various studies regard the incidence of orbital blowout fractures as highest in young adults, especially those aged 20–29 years [54,55,56].

A significant impact on relative orbital volume change depending on the trauma mechanism was not found in this study (*p* = 0.101). However, the trauma mechanisms, listed from most common to least common, were assault, fall, sports-related accident, traffic-related accident, and others. Assaults, falls, and traffic accidents being the most frequent causes of blowout fractures are supported in other studies as well [54,55]. Tong et al. [57] suggested that pure orbital blowout fractures were most strongly associated with assaults, but orbital fractures, such as impure blowout fractures, were more commonly associated with motor vehicle accidents. Another study found that trauma mechanisms are related to fracture type; isolated orbital floor accidents were most frequently caused by blunt object hits, traffic accidents, assaults, and falls, whereas combined fractures, including several fractured sites, were mostly caused by sports accidents, assaults, and falls [13].

Giovannetti et al. [58] reported in their study that during the COVID-19 pandemic there was a significant decrease in facial fractures and that the leading cause during this period was domestic accidents, followed by assaults. Traffic accidents and sports accidents were the most common trauma mechanisms during the time before and during COVID-19. Our study includes patients both before and after the pandemic, but the difference in trauma mechanisms is still notable. Whang et al. found that the number of blowout fractures decreased, especially in patients aged under 20 years, but that blowout fracture surgery, on the other hand, increased in patients older than 60 years [59]. Another study stated that the proportion of blowout fractures in ophthalmologic consultations increased during the pandemic [60].

No significant difference in relative orbital volume change was found between females (mean 3.59) and males (mean 4.92). However, a wider dispersion was observable in males; the range of the relative volume change was 81.3, whereas in females, it was 50.6. Earlier studies have concluded that young males have a higher risk for orbital fractures [55,61,62]. According to Khojastepour et al. [56], this could stem from the fact that men are more often involved in traffic accidents, situations with physical assault, and occupations with higher risks for facial trauma. When examining the relative volume change with age and trauma mechanism, we simultaneously found that trauma mechanisms differ a lot depending on the patient’s age. For adults, the main mechanisms for relative volume change in fractures of the bony orbit are physical assault and traffic accidents. For geriatric patients, falls are notably more common. These findings are supported by earlier studies as well [6,62]. Su et al. [47] found that, in underaged patients, the most common trauma mechanism is traffic accidents.

The retrospective nature of the study may slightly distort the obtained results because it is possible that minor non-operative fractures with minimal clinical findings may not necessarily be registered in a primary evaluation. Thus, the diagnosis code is not found in the patient registry, although a fracture may have been detected by a senior radiologist in re-evaluation of the image during office hours. It must also be kept in mind that analysing three-dimensional volumes from CT images may involve a marginal error.

Possible drawbacks of the study were that the duration of the patients’ double vision was not measured or compared to the volume change. Also, the fracture site was not specified for a certain area in the bony orbit. The strengths of this study were that PHCH is the central hospital of its area, which means that all fractures of the bony orbit are sent there for treatment, except for extremely severe cases, which are forwarded to a tertiary hospital. In addition, the data is collected over a long period of time, and the orbital volume has been measured with an automated segmentation tool to prevent errors caused by manual volume measurement. All cases have thereafter been controlled by the same radiologist (N. L.).

## 5. Conclusions

In conclusion, we examined the association between relative orbital volume change in blowout and blow-in fractures with respect to age, gender, and trauma mechanism. We found a statistically significant association between an increase in relative volume change and increase in age. Trauma mechanism and sex did not affect the relative volume change; however, the age profile differs in males and in females as well as in trauma mechanisms.

## Figures and Tables

**Table 1:** Descriptive statistics of the variables.

	Mean	95% CI		Range	Distribution Sig.
Age (years)	48.2	44.5	51.8	9.90 to 91.4	0.004
Volume change%	4.39	2.63	6.15	−16.0 to 65.3	<0.001
	Females (%)	Males (%)			
Sex	51 (40.2)	76 (59.8)			
	Fall (%)	Assault (%)	Sports (%)	Traffic (%)	Others (%)
Trauma mechanism	44 (34.6)	56 (44.1)	12 (9.4)	11 (8.7)	4 (3.1)

CI, confidence interval.

**Table 2:** Comparison of variables to orbital volume change. * *p* < 0.05, significant.

	Number of Groups	Degree of Freedom	Correlation Coefficient	Significance
**Age**			0.204	*p* = 0.022 *
**Sex**	2			*p* = 0.535
**Trauma mechanism**	5	4		*p* = 0.101

**Table 3:** Comparison of variables to age. * *p* < 0.05, significant.

	Number of groups	Degree of Freedom	Correlation Coefficient	Significance
**Volume change**			0.204	*p* = 0.022 *
**Sex**	2			*p* = 0.001 *
**Trauma mechanism**	5	4		*p* < 0.001 *

## Data Availability

Non-anonymised data is not available. Anonymised data can be requested from the registry keeper Joint-Authority of health and wellbeing, as determined by the legislation.
